# Possible rifampicin-induced arthritis in a patient receiving anti-tuberculosis therapy: the first reported case

**DOI:** 10.1093/rheumatology/keae272

**Published:** 2024-05-11

**Authors:** Rahaymin Chowdhury, Nawaz Z Safdar, Deborah A B Ellames, Richard J Wakefield

**Affiliations:** Leeds Institute of Rheumatic and Musculoskeletal Medicine, University of Leeds, Leeds, UK; Leeds Teaching Hospitals NHS Trust, Leeds, UK; Leeds Teaching Hospitals NHS Trust, Leeds, UK; Pennsylvania Hospital, University of Pennsylvania Health System, Philadelphia, PA, USA; Leeds Teaching Hospitals NHS Trust, Leeds, UK; Leeds Institute of Rheumatic and Musculoskeletal Medicine, University of Leeds, Leeds, UK; Leeds Teaching Hospitals NHS Trust, Leeds, UK

**Keywords:** rifampicin, arthritis

Rheumatology key message

- Treatment with rifampicin might rarely induce an arthritis.

Dear Editor, R, a 76-year-old male undergoing treatment for pulmonary tuberculosis (TB), was referred to rheumatology by the infectious diseases team with suspected gout secondary to anti-tuberculous therapy. He was diagnosed with polymyalgia rheumatica (PMR) 6 years previously but was lost to follow-up and remained on long term low-dose prednisolone of varying doses. He also had prostate cancer with bony metastases; docetaxel chemotherapy was discontinued 9 months previously after only two cycles. Two days after starting rifampicin, isoniazid and pyrazinamide, R reported pain in multiple small and large joints, which was different in location and more severe to that experienced with his PMR. Prior to referral to us, R’s prednisolone had been increased from 6 mg/day to 15 mg/day but provided little relief. Physical examination revealed swelling over his metacarpophalangeal joints (MCPJ), metatarsophalangeal joints, the left wrist and ankle. Laboratory tests were negative for CCP, RF and ANA antibodies. CRP (25 mg/l) and ESR (59 mm/H) were elevated, with a leucocytosis (neutrophils 12.8 × 10^9^/l, white cell count 14.6 × 10^9^/l) possibly due to prednisolone use. Reduced albumin and haemoglobin were considered secondary to cancer and TB. Elevated serum urate (694 μmol/l) raised the suspicion of pyrazinamide-induced gout and pyrazinamide was ceased. R was discharged on a tapering dose of 20 mg prednisolone in addition to colchicine 500 μg tds. Ten days later, upon review by the rheumatology team, he was reporting ongoing significant pain in his hands, feet and knees alongside milder pain in the neck, right shoulder, groin and lower back despite normalization of serum urate (315 μmol/l). There was no reported improvement of symptoms since pyrazinamide cessation, with no previous history of gout. On examination, joint mobility was severely restricted due to pain. There was marked pitting oedema noted over the dorsum of both hands, MCPJ and ankles. An ultrasound (US) demonstrated low-level grey scale joint synovial thickening with increased power Doppler signal in his wrists and MCPJ (Fig. 1) with similar changes seen around the deep flexor tendon sheaths of the wrist. Marked subcutaneous oedema was also noted over the dorsum of both hands and ankles. There was evidence of calcium pyrophosphate deposition (CPPD) bilaterally in the knee medial menisci but no specific features of gout or rheumatoid arthritis (RA); double contour, tophi or erosions were absent.

**Figure 1.**
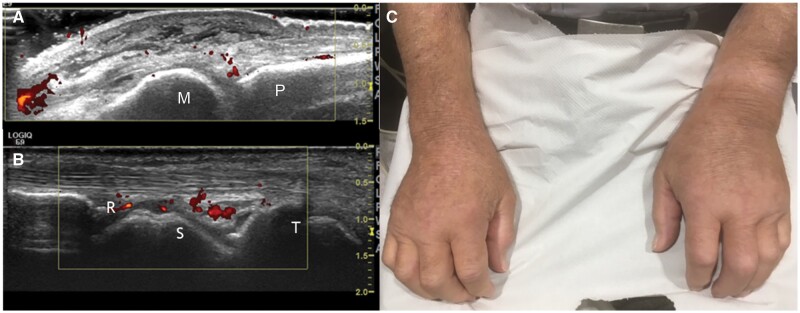
(**A**) Longitudinal dorsal view of a metacarpophalangeal joint. Presence of mild intra-articular synovitis and widespread non-specific extracapsular subcutaneous oedema. M, metacarpal; P, phalanx. (**B**) Longitudinal dorsal view through the radioscaphoid aspect of the wrist on ultrasonography. Presence of mild low-level synovitis with increase power Doppler signal. R, radius; S, scaphoid; T, trapezium. (**C**) R’s hands. Swelling over the dorsum aspect of the hands with marked pitting oedema upon palpation

Two weeks following complete cessation of all anti-tuberculous therapy, R reported significant improvement in joint pains and mobility and managed to walk 2 miles. Treatment for TB resumed sequentially starting with rifampicin and subsequently resulted in widespread return of pain, raising the suspicion of a rifampicin-induced arthritis. Consequently, rifampicin was replaced with pyrazinamide and isoniazid leading to a gradual resolution of arthritis over the following weeks.

## Discussion

Several differential diagnoses were possible in this case. PMR onset RA was initially considered and we questioned whether R may have had RA from the start. However, at the time of the PMR diagnosis, his small joints had not been involved, and he was seronegative and non-erosive. Nonetheless, continued use of steroid was presumed to relate to underlying degenerative joint disease. Alternatively, the PMR had preceded prostate cancer by 5 years; remitting seronegative symmetrical synovitis with pitting oedema/oedema (RS3PE) disease as part of a paraneoplastic syndrome or PMR was considered but deemed unlikely due to the poor response to the steroids [1]. Calcifications noted on the knee US suggested CPPD, but involvement of other joints, most notably the hands, would have been unusual. Polyarticular gout was also considered, but as with CPPD, the distribution was atypical and no evidence was found on US. Finally, given the timing of the symptoms, reactive arthritis secondary to TB was also considered but felt to be unlikely.

At first presentation, arthritis secondary to TB drugs was thought to be the most likely cause. Pyrazinamide is known to increase urate levels [2]; however, R remained symptomatic despite falling urate, cessation of the drug and an absence of features suggesting gout on US. Due to the sparse literature on isoniazid- and rifampicin-induced arthritis, these were not initially considered. According to the British National Formulary, oral rifampicin is not known to cause arthritis but is associated with oedema [3]. A ‘flu-like illness’ including fevers, rigours and headache has been described with intermittent rifampicin use, and rarely, these reported cases include symptoms of arthralgia and myalgia [4, 5]. R did not have a ‘flu-like illness’ and his rifampicin use was not intermittent. Docetaxel, possibly as part of ‘ASIA’ syndrome [6], is also known to cause joint pains and myalgias but symptoms typically resolve within 3 weeks [7]. Finally, although rifampicin has been described as inducing systemic lupus erythematosus (SLE) [8], R did not have clinical or serological features of SLE.

We believe that this is the first reported case of possible rifampicin-induced arthritis outside the context of ‘flu-like illness’ or drug-induced SLE.

## Data Availability

The data underlying this article are available in the article and in its online supplementary material.
